# Electric Bias Induced Degradation in Organic-Inorganic Hybrid Perovskite Light-Emitting Diodes

**DOI:** 10.1038/s41598-018-34034-1

**Published:** 2018-10-25

**Authors:** Bing Xu, Weigao Wang, Xiaoli Zhang, Haochen Liu, Yuniu Zhang, Guanding Mei, Shuming Chen, Kai Wang, Liduo Wang, Xiao Wei Sun

**Affiliations:** 1 Guangdong University Key Lab for Advanced Quantum Dot Displays and Lighting, Shenzhen Key Laboratory for Advanced Quantum Dot Displays and Lighting, Department of Electrical & Electronic Engineering, Southern University of Science and Technology, Shenzhen, 518055 China; 2 Department of Chemistry, Tsinghua University, Beijing, 100084 China; 3 Shenzhen Planck Innovation Technologies Pte Ltd, Ganli 6th Road, Longgang, Shenzhen, 518112 China

## Abstract

For organic-inorganic perovskite to be considered as the most promising materials for light emitting diodes and solar cell applications, the active materials must be proven to be stable under various conditions, such as ambient environment, heat and electrical bias. Understanding the degradation process in organic-inorganic perovskite light emitting diodes (PeLEDs) is important to improve the stability and the performance of the device. We revealed that electrical bias can greatly influence the luminance and external quantum efficiency of PeLEDs. It was found that device performance could be improved under low voltage bias with short operation time, and decreased with continuous operation. The degradation of perovskite film under high electrical bias leads to the decrease of device performance. Variations in the absorption, morphology and element distribution of perovskite films under different electrical bias revealed that organic-inorganic perovskites are unstable at high electrical bias. We bring new insights in the PeLEDs which are crucial for improving the stability.

## Introduction

Organic-inorganic halide perovskite materials, methylammonium lead halide (MAPbX_3_, X = Cl, Br, I) and formamidinium lead halide (FAPbX_3_, X = Cl, Br, I), have attracted much attention for photovoltaic recently, due to high absorption coefficients, long charge carrier diffusion lengths and solution processability^1–5^. In less than five years, the power conversion efficiency of organic-inorganic perovskite (OIP) solar cell increased from 3.8% to 22%^6^. Being excellent light conversion materials, perovskites are also being considered as good light emitting materials, which have tunable band gaps, high color purity, benefit from facile solution processing, and low material cost^7–10^. Researchers made great efforts to improve the photoluminescence quantum yield and the device performance by compositional engineering, processing techniques, and interfacial engineering. The perovskite LEDs (PeLEDs) based on solution-processed method were first demonstrated by Tan *et al*. in 2014, which achieved a luminance of 364 cd m^−2^ and an external quantum efficiency (EQE) of 0.1%^11^. Recently, an EQE of 8.5% was reported for methylammonium lead bromide (MAPbBr_3_)-based green LEDs^12^ and 3.5% for methylammonium lead iodide (MAPbI_3_)-based near-infrared LEDs^13^, which are the highest EQEs reported so far for PeLEDs composed of pure MAPbBr_3_ and MAPbI_3_ films, respectively. More recently, an EQE of 8.8% was achieved for near-infrared LEDs using layered PEA_2_(CH_3_NH_3_)_n−1_Pb_n_I_3n+1_ (PEA = C_8_H_9_NH_3_, phenylethylammonium) perovskite films^14^ and an EQE of 11.7% was achieved by introducing excess large group ammonium halides in the precursor^15^. Despite these high efficiencies, the stability of the device under electric bias has not been well studied.

A well-known weakness of OIP is that they are moisture-sensitive and can degrade to PbX_2_ after exposure to air. Besides material degradation, perovskite solar cell device also suffered significantly from electrical bias in different condition^16–21^. Tomas *et al*. found out that the application of an electric field in inert conditions lead to reversible degradation of perovskite thin film, but in presence of moisture lead to irreversible degradation^16^. Soohyun *et al*. revealed that perovskite solar cell is severely degraded under forward bias (>1 V), even with dark condition^17^. Deng *et al*. monitored the electric field induced both irreversible (>0.4 V μm^−1^) and reversible (<0.4 V μm^−1^) PL response in CH_3_NH_3_PbI_3_ in air with a relative humidity of 40%^18^. Yuan *et al*. presented a reversible conversion between MAPbI_3_ and lead iodide phases under a small electric field (3 V μm^−1^) in air^19^.

Recently, Zhao *et al*. observed an interesting phenomenon that for pure MAPbI_3_ PeLEDs under the influence of subsequent electrical scans, the EQE was improved from an initial 5.9% to 7.4%^22^. This observation is different from what are often observed, where electrical bias/scan generally degrade the device. It was suspected that ion migration is the root cause of this improvement in Zhao’s experiment. There is a pressing need for understanding the mechanisms behind. In this work, electrical bias effect on PeLED has been systematically studied. It is shown that electrical bias can improve or degrade the device performance depending on the magnitude of the bias. By investigating absorbance, SEM surface image and energy dispersive X-ray spectroscopy (EDX) data including point and mapping analysis of perovskite layer before and after electrical bias, the improvement and degradation effect under different electrical bias can be understood.

## Experimental Section

### Synthesis of MAPbBr_3_ Nanocrystals

First MABr was synthesized by mixing the same mole ratio of methylamine (30% in methanol) and hydrobromic acid (48% in water) at 0 °C for 2 h with stirring. The precipitate was collected by evaporation of solvent at 60 °C for 1 h and purified the products by dissolving in ethanol, recrystallizing from diethl ether five times and finally dried in vacuum for 24 h.

For MAPbBr_3_ synthesis, a 200 μL solution containing 0.1 mmol PbBr_2_, 0.15 mmol MABr, 20 μL oleylamine and 500 μL oleic acid in dimethylformamide were added into 10 ml toluene at room temperature to produce the colloidal solution. Acetonitrile was added as a demulsifier in the demulsion process. The green product was collected after centrifugation at 5500 rpm for 4 min, then the precipitates were dissolved into 1 ml n-hexane, a bright green supernatant was obtained after another centrifugation at 5000 rpm for 4 min.

### Fabrication of PeLEDs

MAPbBr_3_ perovskite LEDs were fabricated using ITO patterned glass substrates with a sheet resistance of 15 Ω sq^−1^. ITO substrates were sequentially cleaned using soapy water, deionized water, isopropylalcohol, and acetone in ultrasonicator for 20 min each, and then treated with O_2_ plasma for 30 min prior to film deposition. PEDOT:PSS was spin-coated onto ITO substrates at 3000 rpm followed by thermal annealing at 130 °C for 20 min in air. Then these ITO substrates were transferred into N_2_ glove box for further use. TFB in chlorobenzene was spin-coating on top of PEDOT:PSS at 4000 rpm, baked at 120 °C for 20 min. Perovskite were deposited at 2000 rpm without any annealing. Finally, TPBi, LiF and Al were deposited using thermal evaporation at rate of 1.0, 0.1 and 1.0 nm s^−1^, respectively, under a high vacuum (<1.5 × 10^−6^ Torr). The device area was 2 × 2 mm^2^.

## Characterization

The EL spectra of the PeLED were measured with a fiber optic spectrometer (Ocean Optics USB2000). Device performance was achieved consisting with a calibrated PIN-25D silicon photodiode, a dual-channel Keithley 2614B source measure unit. Light emitted from PeLEDs was absorbed by the photodiode, which had a known responsivity and was measured as photodiode current, which were monitored by Keithley source. Then these two quantities were used to calculate the EQE. Surface image and element analysis were measured by using an FE-SEM (Merlin, Carl Zeiss). The XPS measurements were performed by ESCALAB 250Xi (Thermo Fisher).

### Parameters for device measurement

Voltage range: 0 V to 5 V.

Step: 0.2 V.

Pulse time (each point): 100 ms.

Electrical bias was conducted by Keithley 2400 with constant voltage (0 V, 3 V, 4 V, 5 V, 6 V) in 60 s. The device performance was measured every 10 s during the electrical bias.

**Figure 1:**
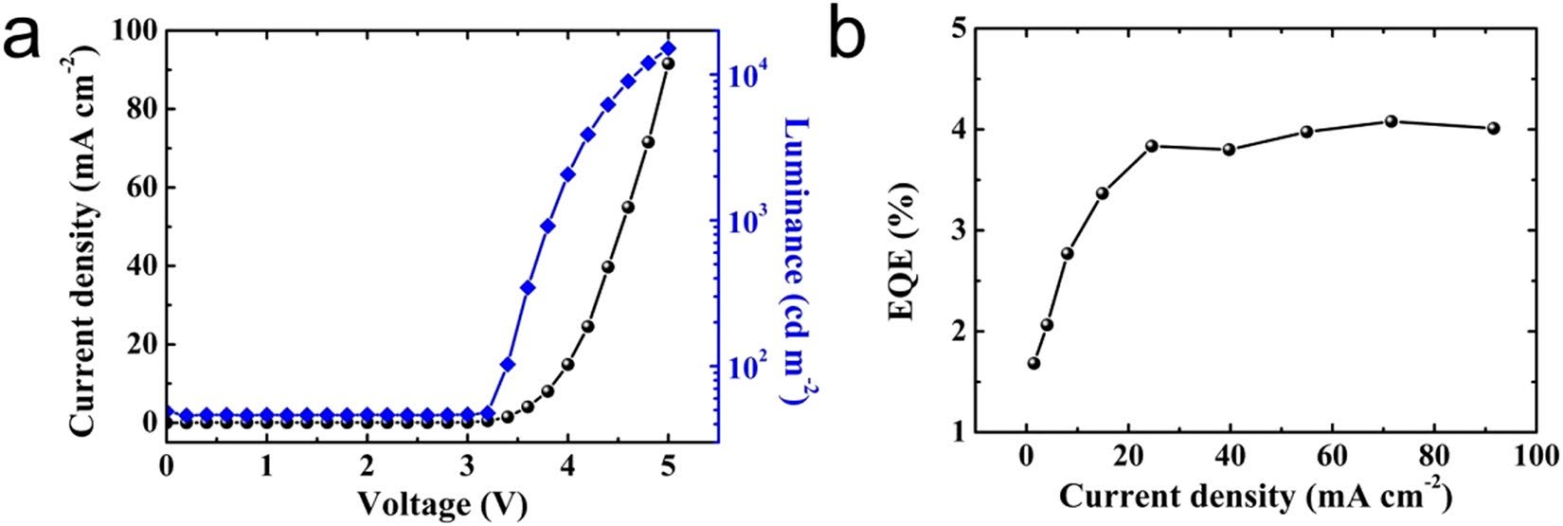
(**a**) Current density and luminance versus voltage and (**b**) EQE versus current density of PeLED measured after fabrication.

Perovskite nanocrystal is synthesized based on the protocol reported in the literature^23^. And the PeLEDs were fabricated with a device structure of ITO/PEDOT:PSS/TFB/MAPbBr_3_ perovskite/TPBi/LiF/Al shown in Fig. S1a, along with a schematic energy diagram of the device in Fig. S1b, where PEDOT:PSS and TFB are serving as a hole injection layer and hole transporting layer respectively, TPBi as electron transporting layer. The current density *vs* voltage curve is shown in Fig. 1a, which is clearly a rectifying curve of a diode. The corresponding luminance and external quantum efficiency curve is shown in Fig. 1a,b, with a maximum luminance of 15000 cd m^−2^ and a EQE of 4% at 91.6 mA cm^−2^, revealing a relatively good performance. The roll-off started at 8 V, which is no significant break down during our measurement (Fig. S2, Supporting Information).

**Figure 2:**
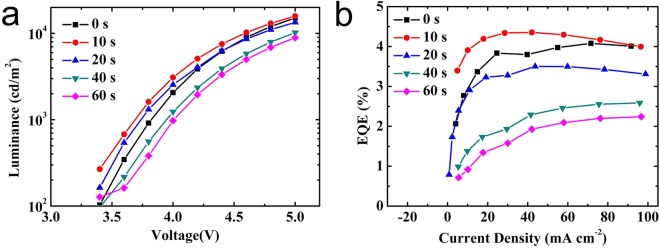
(**a**) Luminance versus voltage and (**b**) EQE versus current density of PeLED with subsequent scans.

Interestingly, we find that the luminance and EQE first increase and then decrease after several electrical scans (Fig. 2a,b). As shown in Fig. 2b, EQE increased at the second measurement, then decreased after the third measurement. But there is little change in J-V curves, as shown in Fig. S3, which indicates low emitting efficiency of PeLED. The enhancement under low current density is due to the motion of excess ions^22^, like excess MA or bromide in perovskite nanocrystals fill vacancies and reduce the defects, but under high current density, the degradation of perovskite film leading to more defects, which in turn decrease device performance. The same phenomenon also happens in luminance (Fig. 2a).

**Figure 3:**
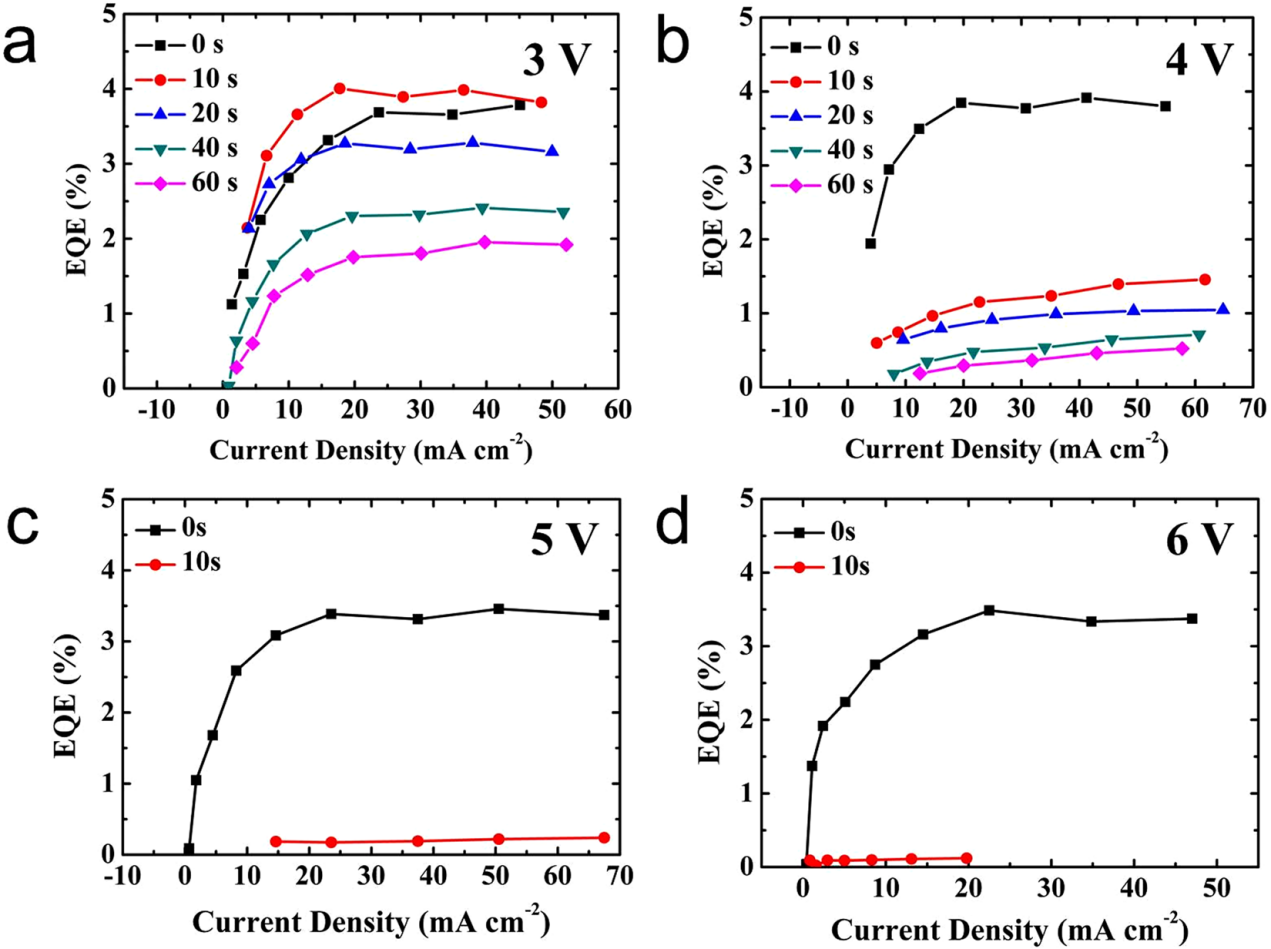
EQE versus current density of PeLED under different electrical bias (**a**) 3 V, (**b**) 4 V, (**c**) 5 V, (**d**) 6 V.

In order to understand the reason behind this decrease in performance, we have applied different electrical biases to the PeLEDs (Fig. 3). Figure 3a shows the variation curve of EQE under 3 V bias for one minute. The EQE of PeLED under 3 V bias has the same trend as device without any bias. The EQE can be improved almost 20% at 15 mA cm^−2^ after 3 V 10 seconds bias, but decreases with continues bias. After applying the bias for one minute, the EQE decreased by 51% at the current of 50 mA cm^−2^. The variation curves of EQE under 4 V, 5 V and 6 V biases are different from that of 3 V bias, which shows only decay with continue electrical bias. Under 4 V continues bias (Fig. 3b), the EQE are dramatically decreased from 3.8% to 1.4% within 10 s bias stress, then decreased to 0.5% after another 50 s. The PeLED devices immediately failed under 5 V and 6 V bias stress for 10 s.

**Figure 4:**
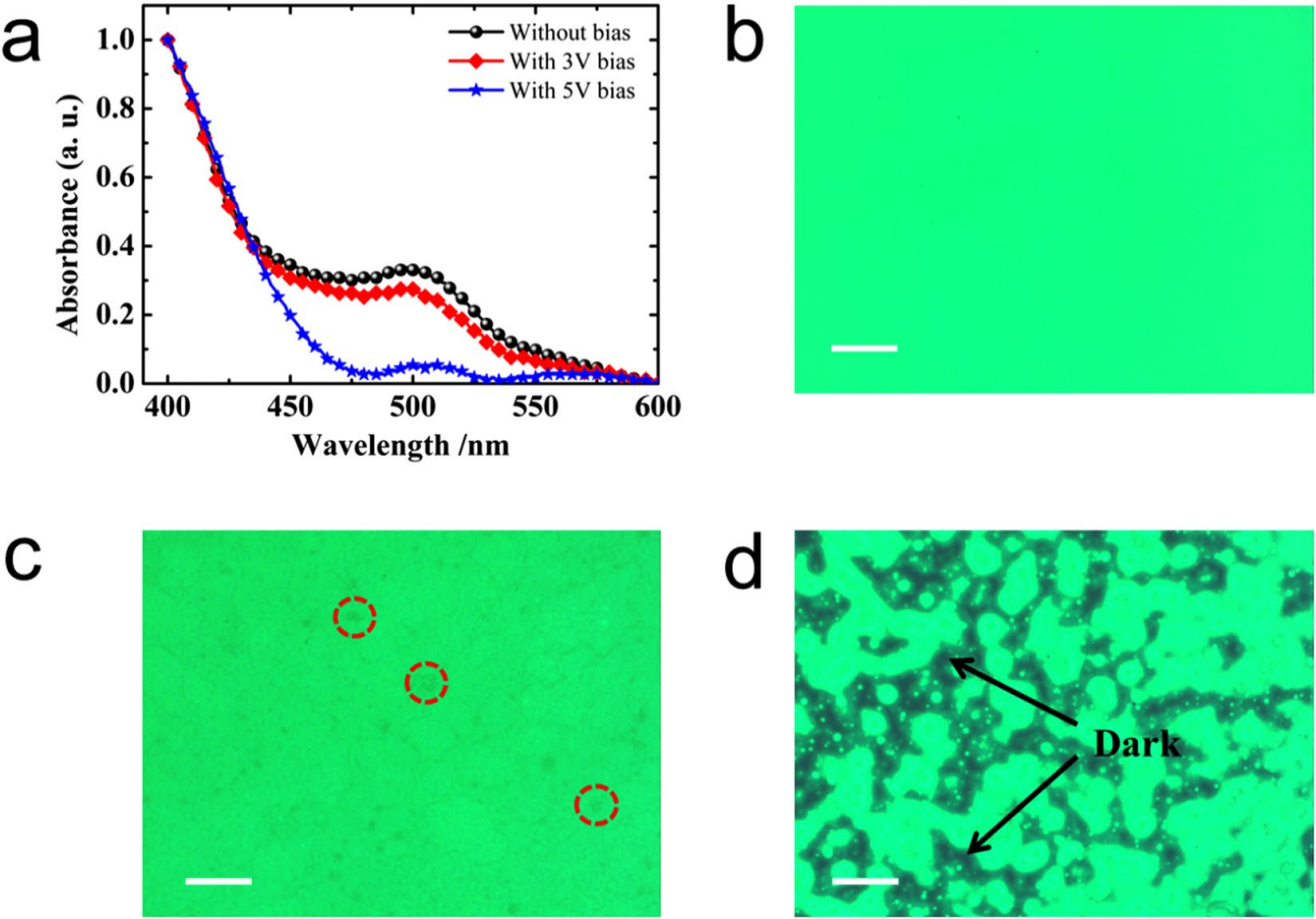
(**a**) Absorbance of perovskite film under different electrical bias and images of perovskite film under fluorescence microscope, (**b**) 0 V, (**c**) 3 V, (**d**) 5 V. (The structure of the samples were ITO/PEDOT:PSS/TFB/perovskite, LiF/Al was removed by tape. The scale bar is 100 μm).

To identify the degradation products left behind after the electrical bias, we further characterize the perovskite film by measuring absorption and fluorescence microscope. In Fig. 4a, we plot the absorption of perovskite film after different electrical bias. As electrical bias increases, we observed a reduction in absorption which means the degradation of perovskite film. The fluorescence microscope images indicate that the emission of perovskite film under UV light is relative uniform without electrical bias. After electrical bias, perovskite films start to degrade which shows that there is no emission for some areas sparsely distributed across the area examination. To further confirm the degradation of perovskite film, X-ray diffraction was performed, which observed the decrease of peaks intensity for perovskite crystals (Fig. S4a). And after the electrical bias, the diffraction peak of PbBr_2_ appears (Fig. S4b), which means PbBr_2_ is one of decomposed products.

**Figure 5:**
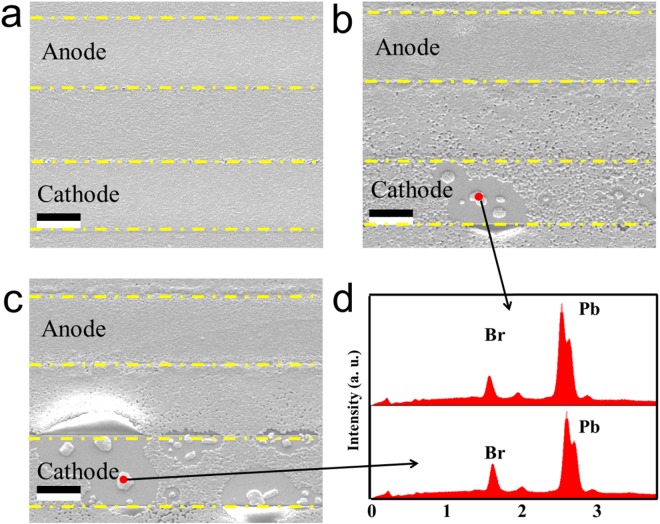
SEM images of perovskite film under different electrical bias in 60 s, (**a**) without electrical bias, (**b**) 3 V, (**c**) 5 V and (**d**) EDX point analysis. (The scale bar is 10 um).

To further investigate the degradation mechanisms of perovskite film under electrical bias, we analyzed EDX elements distribution under different electrical bias. Figure 5 displays the morphology of perovskite film driven in one minute at three different voltages (0, 3, and 5 V). As seen in Fig. 5a, the perovskite film has smooth morphology. Significant islands start to appear after reaching 3 V bias in one minute, and the number of these islands grows with the increase of the electrical bias accordingly. In order to find out the elemental composition of the island, we conducted the EDX point analysis, shown in Fig. 5d, which contains Pb and Br only. But C, N, Pb and Br can be detected in perovskite film before electrical bias, shown in Fig. S5. And XPS results (Fig. S6) show that there is no Pb metal peak after electrical bias. So combining with the XRD, XPS and EDX results, the possible mechanism of degradation is shown as follows^19,24^:

$$\begin{array}{c}{{\rm{CH}}}_{3}{{\rm{NH}}}_{3}{{\rm{PbBr}}}_{3}\to {{\rm{CH}}}_{3}{{{\rm{NH}}}_{3}}^{+}+{{\rm{PbBr}}}_{2}+{\text{Br}}^{-}\\ \,\,\,\,2{{\rm{Br}}}^{-}\to {{\rm{Br}}}_{2}\uparrow +2{e}^{-}\end{array}$$

**Figure 6:**
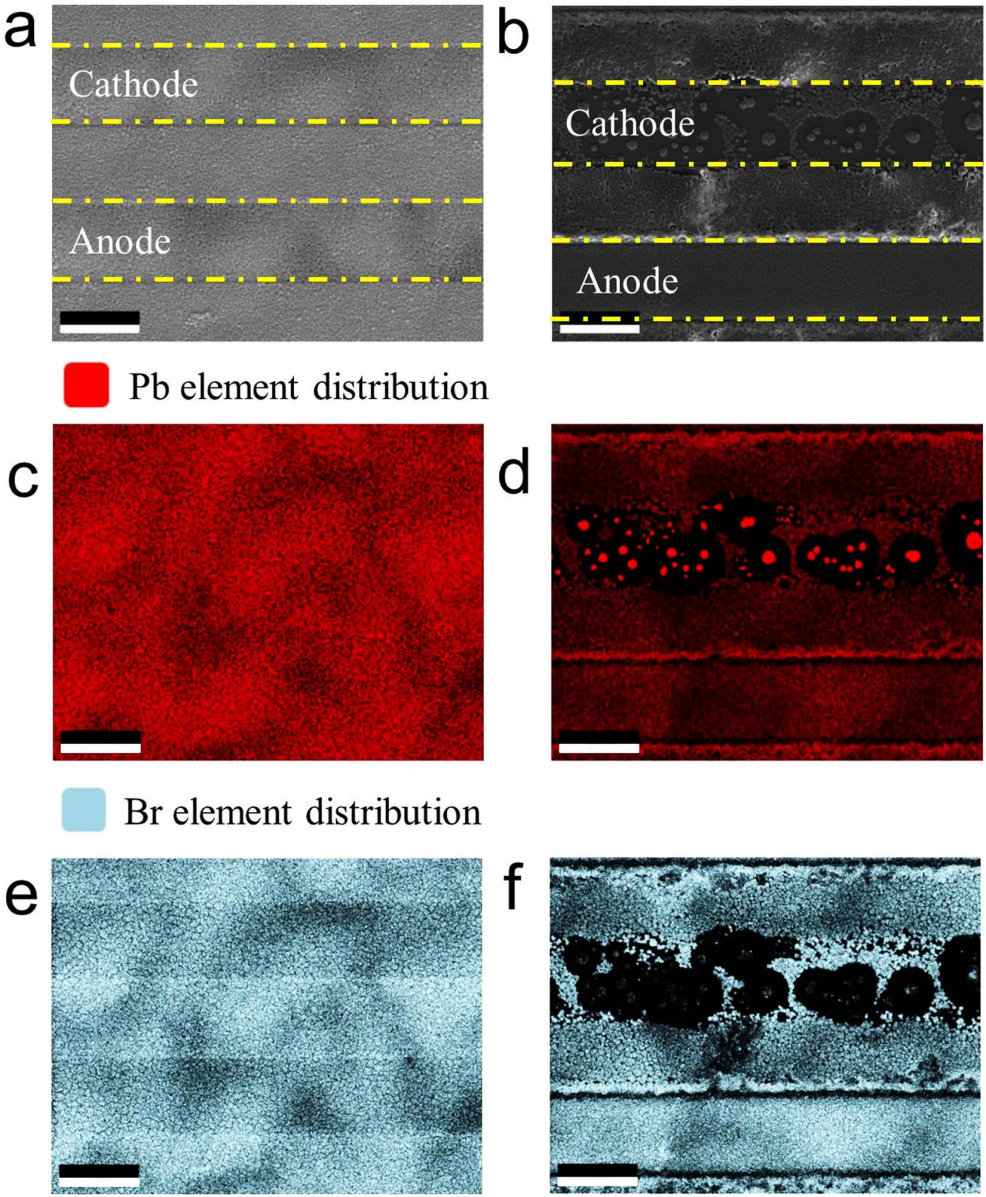
EDX elements mapping profile of Pb, Br after 0 V and 5 V bias. (**a**) and (**b**) are the corresponding image, (**b**,**c**,**e**,**f**) are Pb and Br distribution, respectively. (The scale bar is 20 um).

This degradation will lead to breakdown of the perovskite film, which is responsible for the decay in device performance. In order to further understand Pb and Br elements distribution, EDX mapping was conducted, as shown in Fig. 6. Before any electrical bias is applied, Pb and Br are evenly distributed across the whole perovskite film. When 5 V electrical bias is applied, leading to unevenly distribution of Pb and Br.

In conclusion, using perovskite light emitting diodes with EQE as high as 4%, we studied the degradation in PeLEDs under different electrical bias. To observe degradation induced by electrical bias, biasing tests were conducted under different voltage. Under low electrical bias, the efficiency can be improved, owing to the excess ions (like Br^−^) filling vacancies. On the other hand, under long time operation or high electrical bias, the performance will immediately decay, mainly due to degradation of the perovskite film. Additionally, the degradation rate of perovskite depends on the magnitude of the forward electric bias. This work highlights the necessity of further research to improve the stability of PeLEDs.

## Electronic supplementary material

Supplementary Information
