# Supplementary material for: Electric Bias Induced Degradation in Organic-Inorganic Hybrid Perovskite Light-Emitting Diodes
Source: Sci Rep. 2018 Oct 25;8:15799. doi: 10.1038/s41598-018-34034-1 (PMC6202315; doi:10.1038/s41598-018-34034-1)
Supplement: Supplementary file 1 — Supplementary Information [file 41598_2018_34034_MOESM1_ESM.docx]

Supporting Information

Electric Bias Induced Degradation in Organic-Inorganic Hybrid Perovskite Light-Emitting Diodes

Bing Xu †^a^, Weigao Wang †^a^, Xiaoli Zhang ^a^, Haochen Liu ^a^, Yuniu Zhang ^a^, Guanding Mei ^a^, Shuming Chen ^a^, Kai Wang ^a^, Liduo Wang ^b^, Xiao Wei Sun ^a^

^a^ Department of Electrical & Electronic Engineering, Sourthern University of Science and Technology, Shenzhen, 518055, China

^b^ Department of Chemistry, Tsinghua University, Beijing 100084, China


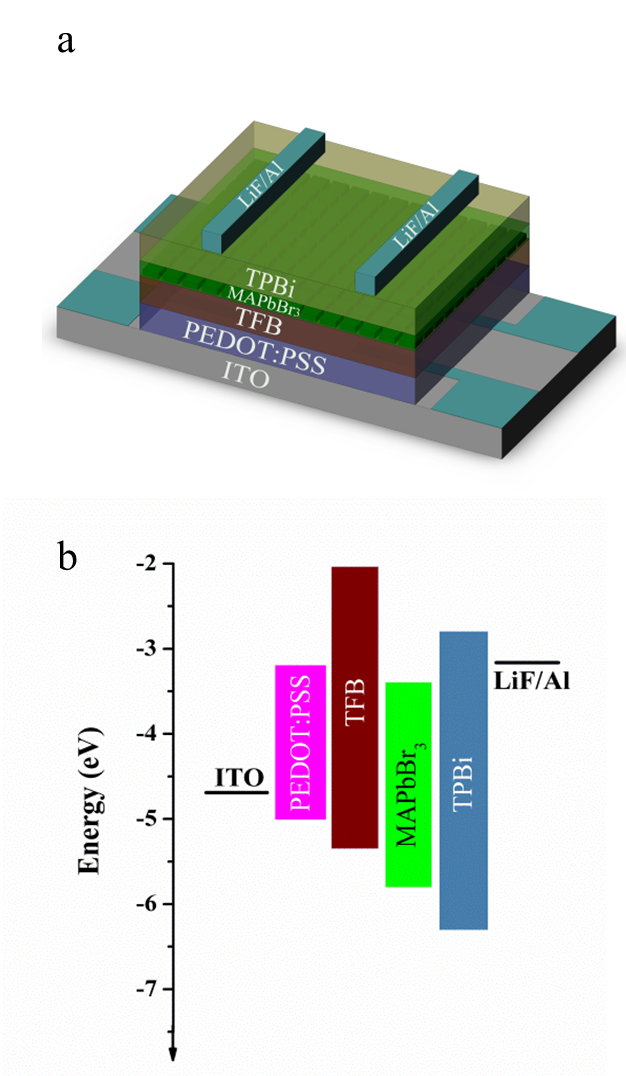


Figure S1 PeLED device structure and energy diagram of each layer





Figure S2 Luminance start to roll off at high voltage (8V)





Figure S3 J-V curve of multiple electrical scan


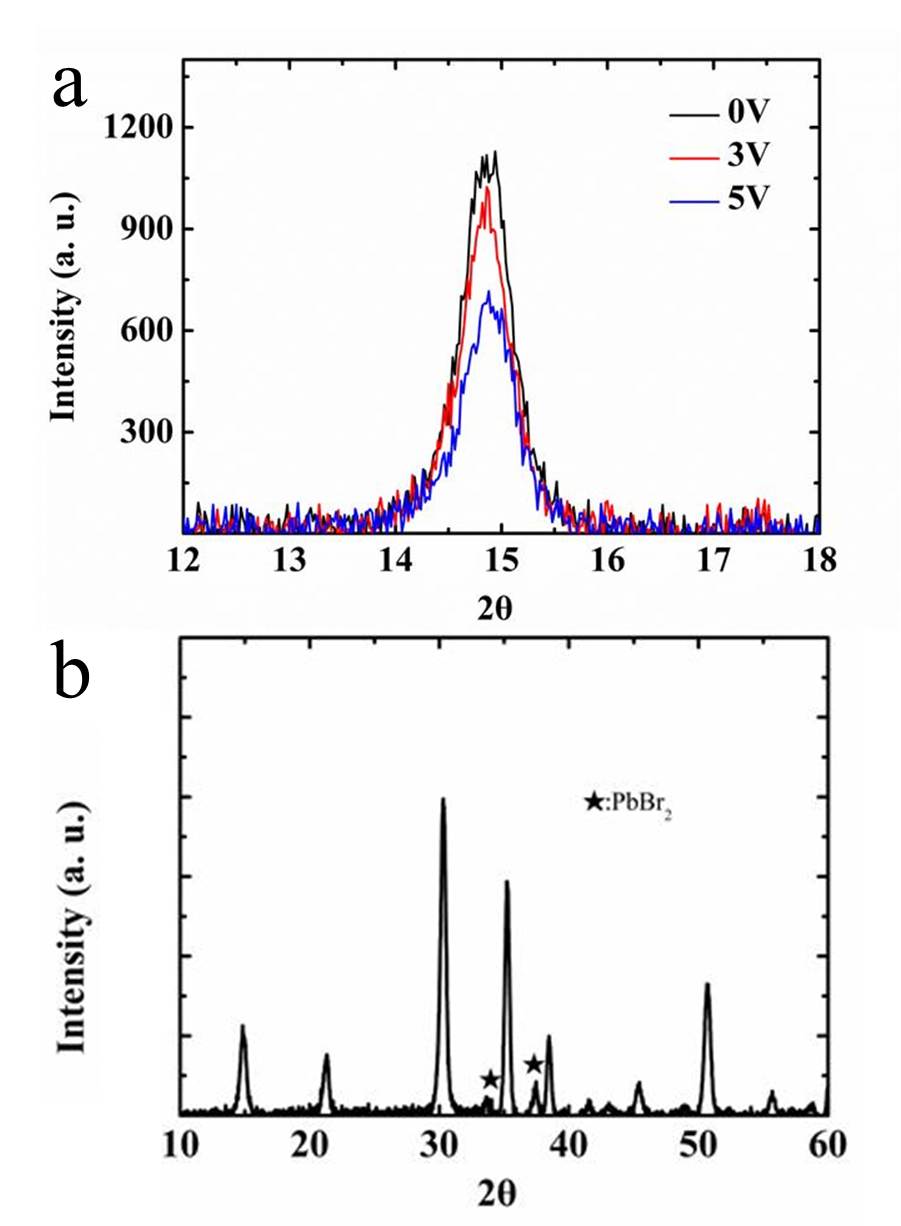


Figure S4 a) XRD pattrens of perovskite LED device under different electrical bias, b) PbBr_2_ peaks appear after electrical bias.


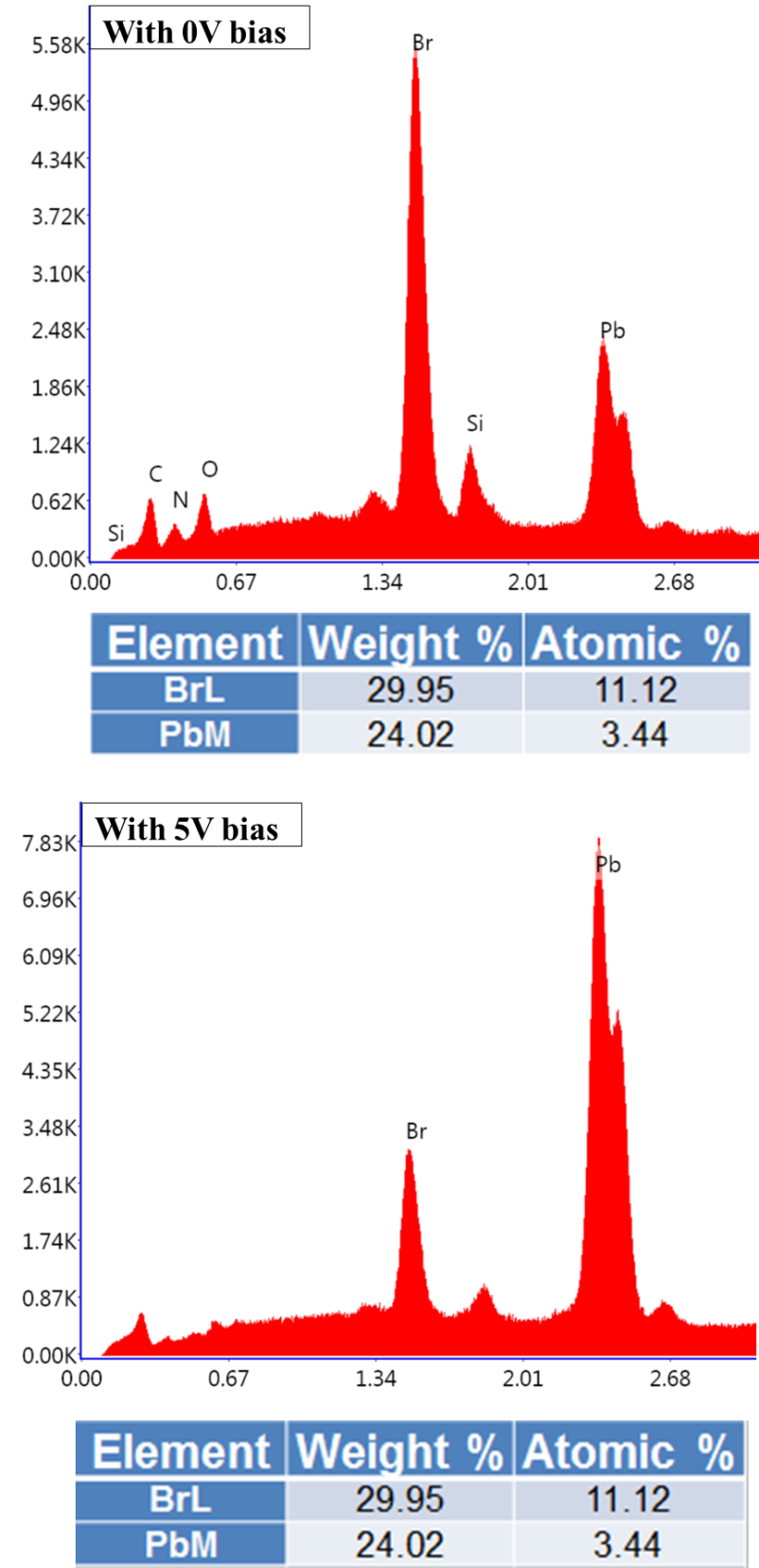


Figure S5 EDX point analysis





Figure S6 X-ray photoelectron spectroscopy (XPS) analysis of Pb element
